# Mechanism for Utilization of the *Populus*-Derived Metabolite Salicin by a *Pseudomonas*—*Rahnella* Co-Culture

**DOI:** 10.3390/metabo13020140

**Published:** 2023-01-17

**Authors:** Sanjeev Dahal, Gregory B. Hurst, Karuna Chourey, Nancy L. Engle, Leah H. Burdick, Jennifer L. Morrell-Falvey, Timothy J. Tschaplinski, Mitchel J. Doktycz, Dale A. Pelletier

**Affiliations:** 1 Biosciences Division, Oak Ridge National Laboratory, Oak Ridge, TN 37830, USA; 2 Genome Science and Technology Program, University of Tennessee, Knoxville, TN 37996, USA; 3 Department of Chemical Engineering, Queen’s University, Kingston, ON K7L 3N6, Canada; 4 Chemical Sciences Division, Oak Ridge National Laboratory, Oak Ridge, TN 37830, USA

**Keywords:** metabolic interactions, *Populus* microbiome, systems biology, multi-omic analysis, *Pseudomonas*

## Abstract

*Pseudomonas fluorescens* GM16 associates with *Populus*, a model plant in biofuel production. *Populus* releases abundant phenolic glycosides such as salicin, but *P. fluorescens* GM16 cannot utilize salicin, whereas *Pseudomonas* strains are known to utilize compounds similar to the aglycone moiety of salicin–salicyl alcohol. We propose that the association of *Pseudomonas* to *Populus* is mediated by another organism (such as *Rahnella aquatilis* OV744) that degrades the glucosyl group of salicin. In this study, we demonstrate that in the *Rahnella*–*Pseudomonas* salicin co-culture model, *Rahnella* grows by degrading salicin to glucose 6-phosphate and salicyl alcohol which is secreted out and is subsequently utilized by *P. fluorescens* GM16 for its growth. Using various quantitative approaches, we elucidate the individual pathways for salicin and salicyl alcohol metabolism present in *Rahnella* and *Pseudomonas*, respectively. Furthermore, we were able to establish that the salicyl alcohol cross-feeding interaction between the two strains on salicin medium is carried out through the combination of their respective individual pathways. The research presents one of the potential advantages of salicyl alcohol release by strains such as *Rahnella*, and how phenolic glycosides could be involved in attracting multiple types of bacteria into the *Populus* microbiome.

## 1. Introduction

*Pseudomonas*, a member of the class *Gammaproteobacteria*, is one of the most common genera colonizing the roots of *Populus*, a woody perennial model system for the study of plant microbe interactions [1,2,3]. Studies have demonstrated *Populus*-altering effects due to the *Pseudomonas*–*Populus* association, including siderophore production, phosphate solubilization, and growth promotion [4,5,6]. Their association with *Populus* might be facilitated by the ability of *Pseudomonas* species to degrade aromatic molecules [7] which are found to be prevalent in the *Populus* metabolome [6].

One class of compounds released by *Populus* is phenolic glycosides (PGs), which have been reported at concentrations of up to 30% of foliar biomass in certain *Populus* clones [8]. PGs consist of a salicin “core” structure attached to various functional moieties such as benzyl-, acetyl- and/or hydroxcyclohexene groups by an esterification process, making salicin the simplest PG [9]. Even though their role as herbivore deterrents is well-studied, information on functions of PGs in shaping the *Populus* microbiome is scarce [10,11,12]. Understanding how the microbiome is influenced by PGs is an important question especially in *Populus* and for that reason, it is important to study how PGs such as salicin are degraded by *Populus* isolates.

While salicin utilization has been observed in some *Pseudomonas* strains [13,14], those particular strains have not been identified in association with *Populus* [6]. Therefore, we hypothesized that another organism within the microbiome might act as a facilitator of the interaction between *Populus* and *Pseudomonas* species. Salicin metabolism has been reported to result in the release of salicyl alcohol [15,16]. Utilization of benzyl alcohol (a compound related to salicyl alcohol) through the naphthalene degradation pathway has been studied in *Pseudomonas putida* CSV86, and the enzymes benzyl alcohol dehydrogenase and benzaldehyde dehydrogenase required for the transformation of benzyl alcohol to benzoate have been characterized [17]. Interestingly, these enzymes also have shown activity against salicyl alcohol and salicylaldehyde [17,18]. In the proposed pathway in *P. putida* CSV86, benzyl alcohol is first transformed to catechol, which is then subjected to ortho-cleavage by way of the β-ketoadipate pathway [18,19,20]. Therefore, we propose that salicin released by *Populus* is metabolized by a salicin-degrading *Populus* isolate, leading to the release of salicyl alcohol, which is utilized by *Pseudomonas* strains.

To test the proposed hypothesis, a salicin-degrading strain was identified from among *Populus* isolates by bioinformatic screening for the salicin utilization gene cluster— *bgl*. Salicin utilization was first described in *Escherichia coli* mutants [21]. The *bgl* operon of *E. coli* responsible for salicin utilization has also been studied in other organisms including *Erwinia chrysanthemi* and *Klebsiella aerogenes* [15,22]. The *bglGFB* gene cluster (Bgl system) encodes proteins BglG, a positive regulator; BglF, phosphoenolpyruvate-dependent sugar phosphotransferase system (PTS) transporter; and BglB, a β-phosphoglucosidase enzyme [23,24]. A similar gene cluster was identified in the *Populus* isolate *Rahnella aquatilis* OV744, which was chosen as the salicin-degrading strain in the present study.

Metabolic interactions between microbial partners are constrained by the niche they inhabit [25]. In fact, studies have implied that hosts such as plants play an active role in the colonization of endophytes by attracting specific bacteria through the release of exudates [26,27,28]. In a natural context, microorganisms interact extensively either through competitive or cooperative means which shape the microbial community of a specific niche [25,29,30,31]. One of the classes of metabolic interactions that shape the microbiome is cross-feeding. D’Souza et al. [32] have classified cross-feeding interactions as either bidirectional or unidirectional based on the reciprocity between the members. Within this classification, unidirectional interactions are deemed unstable [33]. However, cross-feeding of by-products provides a possible mechanism by which stability of unidirectional interactions can be established. Such cross-feeding has been demonstrated in yeast-lactic acid bacteria and in *Bifidobacterium bifidum*-*Bifidobacterium breve* models [34,35].

In the present investigation we utilized bioinformatics, metabolomics, and proteomics to elucidate the molecular mechanism of salicin degradation mediated by *R. aquatilis* OV744 and *P. fluorescens* GM16 co-culture. We demonstrated that *Rahnella* utilizes the sugar moiety of salicin and releases salicyl alcohol, which is subsequently utilized by *P. fluorescens* GM16 using the proposed pathway. The results from this study suggest the presence of a unidirectional cross-feeding interaction between the two organisms. The current research provides insights into potential multi-trophic interactions between *Populus* and its microbiota directly and indirectly through PGs such as salicin. This relationship is potentially important for the establishment and stability of the *Populus* microbiome.

## 2. Materials and Methods

### 2.1. Bacterial Strains and Culture Medium

The γ-proteobacteria *Rahnella aquatilis* OV744 and *Pseudomonas fluorescens* GM16 were previously isolated from *Populus* root tissue [36,37]. The genomes were sequenced and assembled as described elsewhere [37]. The genomes are available in both the National Center for Biotechnology Information (NCBI) [38] (*Rahnella*: GCA_000799835.1; *Pseudomonas*: GCA_000282155.1) and Integrated Microbial Genomes (IMG) [39] (*Rahnella*: 2585427591; *Pseudomonas*: 2511231031) databases. The bacterial strains were maintained on R2A (Franklin Lakes, BD Difco, NJ, USA) agar plates.

For growth assays, individual colonies were picked from the agar plates and grown in liquid R2A medium overnight in the incubator at 25 °C and 250 rpm. The overnight samples were subcultured in 3-(N-morpholino)propanesulfonic acid (MOPS) minimal medium (pH: 7.2) [40] containing the desired carbon source (salicin, salicyl alcohol, or glucose) at concentrations of either 3 mM or 5 mM. Growth was measured using optical density at 600 nm (OD_600_) on a Spectronic 20D+ (Thermo Fisher Scientific, Waltham, MA, USA). The starting OD_600_ of subcultures were between 0.07–0.10. 

### 2.2. Primer Design, DNA Isolation and Microbial Quantification

Cell pellets harvested from bacterial cultures (1:1 starting OD_600_ ratio for co-culture) at different time points during growth were lysed and total DNA was extracted using QIAgen DNeasy Blood and Tissue kit (Qiagen, Hilden, Germany). The DNA was stored at −20 °C for future use. Quantitative PCR (qPCR) was used for detection and quantification of microbes. qPCR was performed using iTaq Universal SYBR Green One-Step Kit (Bio-Rad Laboratories). The primers were designed to be strain-specific for *R. aquatilis OV744* (forward: TGCCTTTCACGCCGATTAT and reverse: TACGGTTACGCACGGTTTC) and *P. fluorescens* GM16 (forward: TGCTTCGGCAGCGATTTA and reverse: CACGGAGATGAAGTTCTCGATAG). The qPCR reaction was run on a CFX96 machine (Bio-Rad Laboratories, Berkeley, CA, USA) and monitored using Bio-Rad CFX Manager v3.1. Bacterial genomic DNA was used as standard for quantification. Following the run, the qPCR data was imported and analyzed in Microsoft Excel as described previously [41]

For determination of the stable state of the co-culture, qPCR was performed on samples over several generations. *Rahnella* and *Pseudomonas* cells were co-cultured in salicin minimal medium. Then, the overnight samples were diluted in the ratio of 1:10 into fresh salicin medium for another growth cycle. This dilution process was repeated for several days and the culture samples were extracted between every dilution. Following the isolation of cell pellets and lysis, qPCR was performed for every sample and the data were analyzed as described previously [41].

### 2.3. Gas Chromatography-Mass Spectrometry (GC-MS) Metabolome Analysis

Secreted metabolites from *Rahnella* and *Pseudomonas* cultured on salicin (3 mM) minimal medium in mono- and co-cultures were determined by GC-MS method. 1 mL of culture supernatants were isolated at different time points during growth and stored at −20 °C. Frozen supernatant samples were thawed and vortexed. 100 μL of sample was transferred to a scintillation vial and 15 μL of sorbitol (1 mg/mL) was added as an internal standard. After drying under a nitrogen stream, the samples were dissolved in 0.5 mL acetonitrile (ACN), and silylated to generate trimethylsilyl (TMS) derivatives, as described elsewhere [42]. After 2 days, 1 μL aliquots were injected into an Agilent 5975C inert XL gas chromatograph-mass spectrometer (GC-MS). The standard quadrupole GC-MS was operated in electron impact (70 eV) ionization mode, targeting 2.5 full-spectrum (50–650 Da) scans per second, as described previously [42].

Metabolite peaks were extracted using a key selected ion, characteristic m/z fragment to minimize integration of co-eluting metabolites. The extracted peaks of known metabolites were scaled back to the total ion current (TIC) using previously calculated scaling factors. Peaks were quantified by area integration and normalized to the quantity of internal standard recovered, sample volume analyzed, derivatized and injected. A large user-created database and the Wiley Registry 10th Edition/National Institute of Standards and Technology (NIST) 2014 Mass Spectral Library were used to identify the metabolites of interest. Unidentified metabolites were represented by their retention time and key m/z ratios.

### 2.4. Sample Preparation for Proteome Measurements

Bacterial strains were grown in triplicates in three different culture conditions in MOPS minimal medium–*R. aquatilis* OV744 in pure culture on glucose or salicin (5 mM), *P*. *fluorescens* GM16 in pure culture on glucose or salicyl alcohol (3 mM), and *Rahnella*-*Pseudomonas* in co-culture on salicin (3 mM). After the cultures reached an OD_600_ of 0.7 or higher, cell pellets were harvested by centrifugation at 11,952× *g* (15 min, 4 °C) and flash frozen in liquid N_2_ and stored at −80 °C. Triplicate samples were processed separately for shotgun proteomics analysis [43]. Cell pellets were suspended in lysis buffer (5% sodium dodecyl sulfate (SDS), 50 mM Tris-HCl, 0.15M NaCl, 0.1 mM ethylenediaminetetraacetic acid (EDTA), 1 mM MgCl_2_, pH 8.5) and lysed by immersing sample tubes in a boiling water bath for 20 min. Chilled (−4 °C) trichloroacetic acid (Fisher Scientific, 99.8%) was added to a final concentration of 25%, and protein was precipitated by storage overnight at −20 °C. Samples were then thawed, and the supernatant discarded. The remaining pellets were washed twice, by vortexing briefly with chilled (−80 °C) acetone, centrifuging at 21000× *g* for 20 min, and discarding the supernatant. The pellets were air dried and then dissolved in 50 mM Tris, 10 mM CaCl_2_ at pH 7.8. For protein denaturation, samples were incubated for 3 h at 60 °C in 6M guanidine, 10 mM dithiothreitol (DTT). Cell lysates were then cooled to room temperature and diluted 6-fold in 50 mM Tris, 10 mM CaCl_2_ at pH 7.8. Trypsin (proteomics grade, Sigma) was added to each lysate at a rate of 20 µg/mg protein and incubated at 37 °C overnight. Then, an identical amount of trypsin was added, followed by incubation at 37 °C for an additional 4 h. The resulting digests were adjusted to 0.1% formic acid (EMD Millipore, Suprapur 98–100%). The removal of particulates and remaining cell debris was carried out by centrifugation through 0.45 μm pore filters (Ultrafree-MC, Millipore, Burlington, MA, USA). Samples were frozen at −80 °C until LC-MS/MS analysis.

### 2.5. Liquid Chromatography/Tandem Mass Spectrometry (LC MS/MS) Analysis

Two-dimensional LC-MS/MS using the multidimensional protein identification technology (MudPIT) approach [44,45] was utilized to analyze the tryptic peptide mixtures. The protocol is described in detail elsewhere [43]. For each growth condition, three biological replicates were analyzed. Using a pressure cell (New Objective, Littleton, MA, USA), digests corresponding to digestion of ~100 μg protein were loaded onto a precolumn fabricated from 150 μm internal diameter (ID) fused silica tubing (Polymicro Technologies, Phoenix, AZ, USA). The precolumn was packed with a 4 cm long bed of reverse-phase chromatographic phase (Jupiter C18, 3 μm particle size, Phenomenex, Torrance, CA, USA) upstream of a ~4 cm bed of strong cation exchange material (5 μm particle size SCX, Phenomenex).

After loading, digests were desalted by attaching the precolumn to an HPLC system (see below) and subjecting to several short reverse-phase gradients to wash away salts, and to elute the peptides from the reverse-phase bed to the SCX bed. The precolumn was then attached to an analytical column through a filter union (Upchurch Scientific, Oak Harbor, WA, USA). Analytical columns were fabricated from 100 μm ID PicoTip Emitters (New Objective) packed with a ~14 cm bed of reverse-phase material (Jupiter C18, 3 μm particle size, Phenomenex, Torrance, CA, USA). The pre- and analytical column assembly was attached to an Accela HPLC system (Thermo Fisher Scientific, Waltham, MA, USA), and two-dimensional LC was effected through application of 11 short-step gradients of increasing salt (ammonium acetate) concentration, each followed by a separate reverse-phase gradient. The eluent was interfaced by a nanospray source (Proxeon) with a linear-geometry quadrupole ion trap mass spectrometer (LTQ-XL, Thermo Fisher Scientific), operating under control of XCalibur software for data acquisition in data-dependent mode. Tandem mass spectra were acquired via collision-induced dissociation (CID) from up to 5 of the most intense ions in each full-scan mass spectrum. Precursor isolation width for CID was 3 *m/z* units, with normalized collision energy set at 35%. Dynamic exclusion was invoked with repeat count of 1, exclusion list size set to 300, repeat duration of 60 s, and exclusion duration of 180 s.

### 2.6. Proteomics Data Analysis

Myrimatch (v. 2.1.138) [46] was used to obtain peptide identifications from tandem mass spectra. IDPicker [47] was used to compile the peptide identifications to determine protein identifications. Protein FASTA files for *Rahnella* (downloaded 16 February 2017 as 2585427591.genes.faa, containing 4962 proteins) and *Pseudomonas* (downloaded 29 June 2016 as 2511231031.genes.faa, containing 5888 proteins) were obtained from the DOE (Department of Energy) Joint Genome Institute Integrated Microbial Genomes (JGI/IMG) database [39], and concatenated with sequences of 44 common contaminant proteins to provide a database for Myrimatch searches. Myrimatch estimated false discovery rates for peptide identification using a sequence-reversed analog of each protein [48,49]. Peptide identification criteria set for Myrimatch required precursor *m/z* tolerance of 1.5, fragment m/z tolerance of 0.5, charge states up to +4, TIC cutoff of 98%, cleavage rule “Trypsin/P”, fully tryptic peptides only, with a maximum of two missed trypsin cleavage sites per peptide. A protein was assumed to be detected if the number of tryptic peptides identified was ≥2 for at least one LC-MS/MS analysis. The false discovery rate for peptide-spectrum matches was set in Myrimatch at ≤2%, and the resulting peptide and protein false discovery rates were ≤0.8% and ≤2.5%, respectively.

Further analyses of proteome data were performed using custom scripts in R [50]. Proteins that shared all their identified peptides were combined into protein groups. Protein abundances were estimated using the spectrum count (number of tandem mass spectra assigned to a protein) [51], corrected for shared peptides [52]. To compare protein abundances across experimental treatments, normalized spectral abundance factors (NSAF) were calculated [53]. For NSAF calculations, adjusted spectrum count values of 0 were replaced with a value of 0.3. NSAFs were calculated in two ways. “Combined” NSAFs totaled 1 for all proteins from both *P. fluorescens* GM16 and *R. aquatilis OV744*, while “by-organism” NSAFs totaled 1 for GM16, and also 1 for OV744. Two approaches were used to identify differentially abundant proteins in pairwise comparisons, based on NSAF. Student’s *T*-tests were performed, assuming unequal variances between two treatments. Benjamini–Hochberg (BH) correction was applied to *t*-test *p*-values. Rank product analysis was also performed, using the RankProd package in R (v. 3.6.0) [54]. The percentage of false prediction (pfp) cutoff of 0.05 (i.e., pfp ≤ 0.05) was applied to evaluate the significance of difference in protein abundance (NSAF values) between two cultures. The fold change for a protein between the two conditions was calculated by determining the ratios of the NSAF values (by-organism), and then log transforming using base 2. For proteins with adjusted spectrum count of zero across all three replicates of a condition, NSAF ratios for pairwise comparisons involving that condition would not be accurate and were tracked as “up” or “down” (i.e., the protein was present in one condition of the pairwise comparison, but not the other) [55].

The proteomics mass spectrometric output files in the original instrument vendor file format, Myrimatch search results, IDPicker analysis files, and the protein sequence file used for searches have been deposited to ProteomeXchange (dataset identifier PXD015876) via MassIVE (dataset identifier MSV000084467).

### 2.7. Bioinformatic Analysis

The tools in JGI/IMG [39], UniProt-based Families (UniFam, Jakarta, Indonesia) [56], and KEGG (Kyoto Encyclopedia of Genes and Genomes) [57] were used for identifying the pathways for substrate metabolism. First, the FASTA files for both strains were extracted from the JGI/IMG database [39], and entered into UniFam genome annotation pipeline [56]. Next, all the possible reactions were determined using the pathway maps in KEGG for all possible substrates/intermediates of salicin or salicyl alcohol pathway in *R. aquatilis OV744* and *P. fluorescens* GM16, respectively. Then, the enzyme commission (EC) numbers for the enzymes catalyzing each reaction were searched against the results obtained from UniFam annotation. Each of the possible genes corresponding to the EC number was then searched in the JGI/IMG server’s “Find Genes” module to corroborate the UniFam annotation. If the EC number could not be matched against the genome annotation, the missing enzyme was predicted based on the operon information because most of the genes involved occurred in operons in both strains. InterPro annotations found in the JGI/IMG server were also utilized for further assurance that the genes encoded for the missing enzymes. Finally, the protein sequences of the enzymes in other set of studies were used as queries against the genomes of *Rahnella* and *Pseudomonas* in *blastp* tool to further confirm the proteins and pathways that are proposed to be involved in the metabolism of salicin and salicyl alcohol, respectively.

## 3. Results

### 3.1. Growth and Bioinformatic Analyses of P. fluorescens GM16 Salicyl Alcohol and Salicin Metabolism

**Figure 1:**
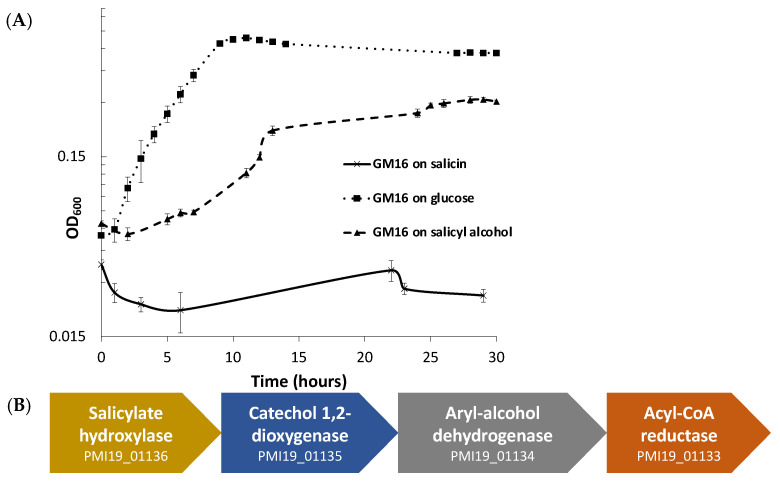
*P. fluorescens* GM16 can utilize salicyl alcohol, but not salicin. (**A**) Growth experiments were performed to determine the substrates P. *fluorescens* GM16 can utilize. As a positive control, glucose was used. *Pseudomonas* was able to grow on glucose and salicyl alcohol minimal media but could not metabolize salicin. (**B**) The genes of the cluster provided in the figure are proposed to encode proteins involved in salicyl alcohol degradation such that salicyl alcohol is oxidized to salicylate first, before being converted to catechol by the action of salicylate hydroxylase. The locus tag numbers for respective genes are prefixed with “PMI19_”.

*Pseudomonas* can utilize and grow on salicyl alcohol as well as glucose minimal medium, but it cannot use salicin (Figure 1A). The growth curve (Figure 1A) demonstrates that there is a considerable lag during the growth of *Pseudomonas* in salicyl alcohol minimal medium. To eliminate the possibility that the growth could be due to the appearance of mutants, an overnight salicyl alcohol subculture of *Pseudomonas* was subcultured in glucose minimal medium before a final subculture in salicyl alcohol minimal medium. The longer lag time persisted even in the final salicyl alcohol culture (Figure S1), which indicates that the lag in growth is due to physiological adaptation, and not because of the emergence of mutants.

For predicting the pathway for degradation of salicyl alcohol, protein sequences of *P. fluorescens* GM16 were annotated using UNIFAM [56] tool followed by comparison of UNIFAM annotations with KEGG [57] database to find the genes that encode proteins for a possible salicyl alcohol degradation pathway. Enzymes that could not be identified in the previous steps were then determined using the protein family annotations and gene cluster structure information in JGI/IMG database. Finally, the protein sequences of benzyl alcohol dehydrogenase, benzaldehyde dehydrogenase, salicylate hydroxylase, and catechol dioxygenase from *P. putida* CSV86 were used for determination of homologs in *P. fluorescens* GM16. This approach led to a gene cluster PMI19_01133–PMI19_01136, which is proposed to be involved in the conversion of salicyl alcohol to muconate using the aforementioned enzymes (Figure 1B). Based on genome-derived knowledge, *P. fluorescens* GM16 is proposed to degrade salicyl alcohol through a catechol intermediate, which is then channeled into the β-ketoadipate pathway. Other proteins involved in the ortho-cleavage pathway of the β-ketoadipate channel were also identified using the process outlined in methods section (Table S1).

### 3.2. Proteomics Analysis of Salicyl Alcohol Grown Cells

To support the proposed mechanism of salicyl alcohol degradation by *P. fluorescens* GM16, a proteomics study on the glucose and salicyl alcohol cultures harvested at the mid-log phase was performed. The comparison of proteome profiles between salicyl alcohol and glucose cultures showed that the predicted proteins involved in the salicyl alcohol pathway were significantly expressed during growth on salicyl alcohol (Table 1). Out of 18 proteins predicted to be involved in the proposed pathway, 14 were detected. In fact, 9 of the predicted proteins were among the 20 proteins with highest increased abundance in salicyl alcohol versus glucose cultures, with their differential abundances all passing our criterion for statistical significance (Rank Product pfp ≤ 0.05 [54]). Other proteins in Table 1 that did not appear to be differentially abundant were either not detected at all, or only weakly observed, with some detected in only salicyl alcohol but not glucose culture. Proteins identified as differentially abundant in salicyl alcohol versus glucose cultures are listed in Table S2; the complete results for all proteins identified in proteome measurements for this study are in Table S10.

For additional support of the proposed salicyl alcohol pathway in *Pseudomonas*, growth assays on the minimal media containing the intermediates of the pathway as sole carbon sources were also performed. *Pseudomonas* was able to grow on salicylate and catechol, further supporting the hypothesized pathway of salicyl alcohol utilization (Figure S2).

### 3.3. Characterization of R. aquatilis OV744 Growth on Salicin

**Figure 2:**
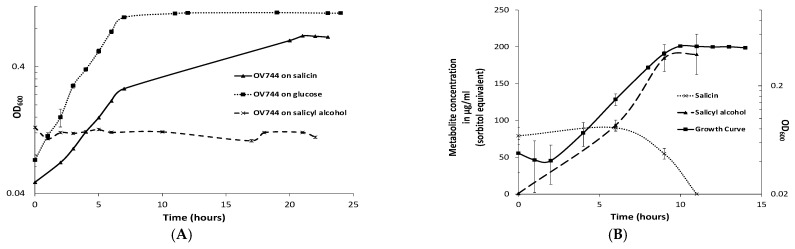
*R. aquatilis OV744* utilizes salicin and secretes salicyl alcohol in the process. (**A**) When *Rahnella* was inoculated with salicin, glucose and salicyl alcohol, it was able to grow on salicin and glucose, but not on salicyl alcohol minimal medium. (**B**) A time-course metabolite analysis was performed on the supernatant of OV744 salicin culture. A reduction in the levels of salicin was clearly visible along with the rise in salicyl alcohol levels in the supernatant samples.

*Rahnella* grew in minimal medium containing salicin or glucose, but not salicyl alcohol (Figure 2A). Using the *E. coli* phosphoglucosidase (glycosyl hydrolase family 1–GH1 which cleaves salicin at its β-glycoside bond) protein sequence, *blastp* was used to predict GH1 homologs in *Rahnella* (Figure S3). Out of five predicted genes, gene EX29DRAFT_02290 (EC 3.2.1.86) was proposed to be the best candidate because the genes flanking it were annotated as a phosphotransferase system (PTS) based transporter/permease (EX29DRAFT_02289) and an anti-terminator regulator (EX29DRAFT_02291) (Table S3). The cluster architecture was similar to the *E. coli bgl* operon. Based on prior knowledge, it was hypothesized that *R. aquatilis OV744* imports and hydrolyzes salicin to glucose 6-phosphate and salicyl alcohol and then uses glucose 6-phosphate for energy generation. In the proposed model, *Rahnella* releases salicyl alcohol into the medium.

To determine whether the predicted pathway for the conversion of salicin was utilized by *Rahnella*, a time-course metabolomic study was carried out. *R. aquatilis OV744* was grown on salicin minimal medium and at different time points, the supernatant was extracted and analyzed using GC-MS. *Rahnella* consumed salicin over time and secreted salicyl alcohol to a final concentration of 190 μg/mL (sorbitol equivalent) (Figure 2B). This experiment provided clear evidence that *R. aquatilis OV744* only utilizes the glucosyl moiety of salicin and secretes salicyl alcohol into the medium.

### 3.4. Proteomics and Metabolomic Investigation of Salicin Utilization

To support the annotation-based prediction that the *bgl* operon is present in *Rahnella* for salicin utilization, a proteomics study was performed. In the study, the protein expression profiles between cells grown on salicin versus glucose and harvested at mid-exponential phase were compared. The proteins hypothesized to degrade salicin, PTS transporter/permease (EX29DRAFT_02289) and 6-phospho-β-glucosidase (EX29DRAFT_02290), were detected in salicin cultures but were absent in all the replicates in glucose cultures (Table 2). The protein encoded by the anti-terminator gene (EX29DRAFT_02291) was not detected in either salicin or glucose culture. Proteins identified as differentially abundant in salicin versus glucose cultures are listed in supplementary Table S4; the complete results for all proteins identified in proteome measurements for this study are in Table S10.

Metabolomics was performed to identify the intermediate metabolites of the proposed pathway. PTS-based transporters are known to phosphorylate their substrates, which means salicin should be imported as salicin 6-phosphate. Spectral data from GC- MS analysis of both intracellular and secreted metabolites were analyzed. Salicin 6-phosphate was not detected in the supernatant samples of salicin monocultures.

### 3.5. Analysis of R. aquatilis OV744 and Pseudomonas sp. Co-Culture Unidirectional Cross-Feeding Mechanism

**Figure 3:**
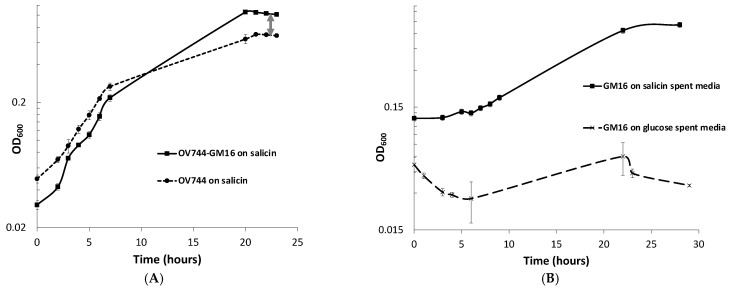

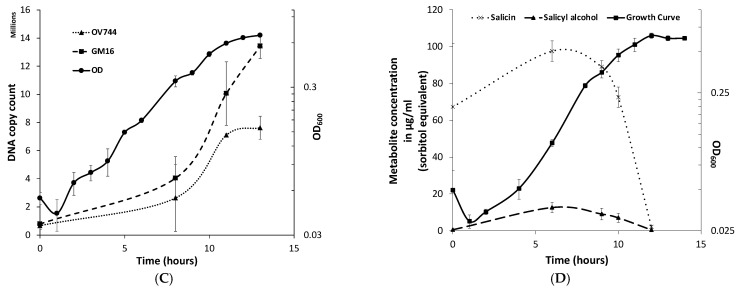
*R. aquatilis* OV744 and *P. fluorescens* GM16 can both grow on salicin co-culture by cross-feeding salicyl alcohol. (**A**) When Rahnella was co-cultured with *Pseudomonas* on salicin, a spike in OD600 (visualized by double-arrowed line) was observed compared to the OD_600_ of *Rahnella* salicin monoculture. (**B**) *Pseudomonas* was inoculated with *Rahnella*-grown salicin and glucose spent media. *Pseudomonas* was not able to utilize the spent medium from glucose culture, but it could use spent medium from salicin culture. (**C**) A qPCR experiment was performed to monitor the growth of individual strains in salicin co-culture. As the co-culture grew (as monitored by OD_600_), both *Rahnella* and *Pseudomonas* were able to multiply as demonstrated by the increase in the DNA copy count of both strains. (**D**) A time-course metabolite analysis on the supernatant of salicin co-culture demonstrated decrease in salicin levels over time. The salicyl alcohol levels remained low throughout this time suggesting secreted salicyl alcohol utilization by *Pseudomonas* in co-culture.

When monitoring the growth of *Rahnella*-*Pseudomonas* co-culture in salicin minimal medium, a spike in OD_600_ compared to that of *Rahnella* monoculture was observed (Figure 3A). Since *Rahnella* can only utilize salicin, releasing salicyl alcohol, while *Pseudomonas* can only degrade salicyl alcohol, it was hypothesized that the higher OD_600_ was observed due to the growth of both strains in salicin such that *R. aquatilis OV744* cross-feeds salicyl alcohol to *Pseudomonas*. To test this hypothesis, an experiment was performed to determine if *Pseudomonas* could grow on spent media of *Rahnella* salicin cultures. Growth was observed on salicin-spent medium, but not on glucose-spent medium–negative control (Figure 3B).

qPCR experiments were also performed to quantify the growth of both strains on salicin co-culture. The qPCR data clearly confirmed that both strains were able to grow on salicin co-culture as the DNA copy count of each strain increased over time (Figure 3C, Table S5). Remarkably, it was discovered that the final gene copy count of *Pseudomonas* was higher than that of *Rahnella* even though *R. aquatilis OV744* is the provider in the predicted cross-feeding interaction. Since it was an unusual phenomenon, another set of qPCR experiments to determine the stable state of the co-culture was carried out. In this case, the growth of both strains in the co-culture in salicin minimal medium was monitored over several generations. The results from this experiment demonstrated that the growth of *Pseudomonas* takes over the growth of *Rahnella* in co-culture. In fact, the ratio between *Pseudomonas* and *Rahnella* DNA copy count in the co-culture was consistently at the ratio ~2:1 over ten generations (Figure S4, Table S6). The comparison of DNA copy count curves between the salicin monoculture (Table S7) and co-culture (Table S5) for *Rahnella* also suggests that *Rahnella* does not grow as well in co-culture as it does in monoculture (Figure S5).

To determine the nature of interaction between *P. fluorescens* GM16 and *R. aquatilis OV744* in salicin co-culture, more experiments were performed. Association of *Pseudomonas* to *Rahnella* does not appear to provide any growth advantage to *Rahnella*, because salicyl alcohol did not inhibit growth of *Rahnella* appreciably at concentrations as high as 3 mM (Figure S6). Similarly, experiments were performed to determine whether secreted metabolites of *P. fluorescens* GM16 were able to inhibit the growth of *R. aquatilis OV744*. Neither the spent medium of the salicyl alcohol culture, nor that of the salicin co-culture, inhibited the growth of *Rahnella* (Figure S7).

### 3.6. Metabolomics and Proteomics Data Analysis of Metabolic Cross-Feeding Interaction between R. aquatilis OV744 and P. fluorescens GM16

For the confirmation of the cross-feeding of salicyl alcohol between the two strains, a time-course metabolite study was performed on the supernatant of the co-culture. *Rahnella* and *Pseudomonas* were grown together on salicin minimal medium, and supernatant samples were extracted at different time points during growth. GC-MS analyses of the supernatant samples demonstrated that over time, salicin was utilized by the co-culture, but salicyl alcohol levels remained negligible (0–12 μg/mL, sorbitol equivalent) throughout (Figure 3D). This experiment further supported the hypothesis that *Rahnella*-secreted salicyl alcohol is utilized by *Pseudomonas* in co-culture.

**Figure 4:**
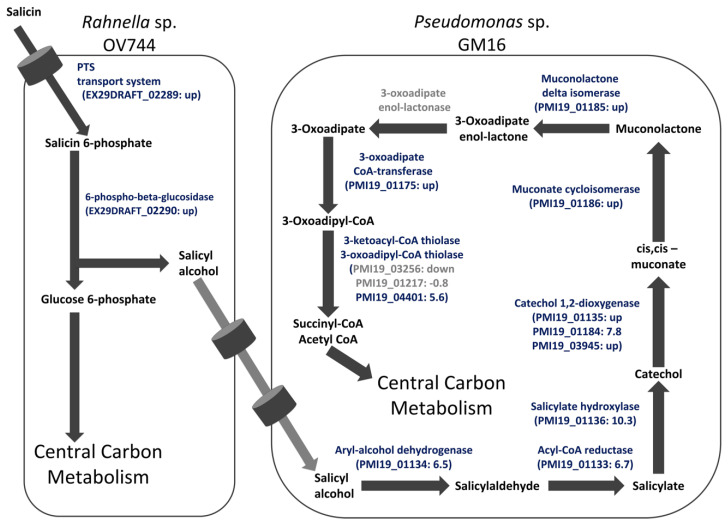
Proteomics data support the salicyl alcohol cross-feeding hypothesis between *R. aquatilis OV744* and *P. fluorescens* GM16 in the salicin co-culture. Proteome data from the *Rahnella*-*Pseudomonas* salicin co-culture were compared to data of glucose monocultures of each strain. For *Rahnella*, proteins of the proposed salicin degradation pathway were upregulated in co-culture compared to glucose monoculture. Similarly, protein expression of *Pseudomonas* was higher for proteins in co-culture for the proposed salicyl alcohol metabolic pathway than those in glucose monoculture. Values within parentheses represent log_2_ ratio of fold change of NSAF values. In cases where the protein was detected in only one culture, the “up” or “down” are reported instead of a fold change. Proteins passing the pfp cutoff are in blue whereas those that did not pass the cutoff are in grey. Some predicted proteins that were not identified as differentially abundant (Tables S8 and S10) are omitted from this figure. For 3-oxoadipate enol-lactonase, none of the proteins were detected reliably in either co-culture or glucose culture. The light grey arrows represent yet to be determined transporters of salicyl alcohol.

To further corroborate the salicyl alcohol cross-feeding hypothesis and to determine whether the proteins detected in monocultures were also present in salicin co-culture, a study comparing the proteomes of *Rahnella* and *Pseudomonas* glucose monocultures and salicin co-culture was carried out. For both *Rahnella* and *Pseudomonas*, most of the proteins of the proposed salicin and salicyl alcohol pathways, respectively, were only detected in co-culture or were significantly more abundant in co-culture compared to in glucose culture (Table S8). In the case of *Pseudomonas*, none of the proteins encoding 3-oxoadipate enol-lactonase were reliably detected in either glucose culture or co-culture (Table S10). Overall, the proteomic study further established the proposed model of unidirectional salicyl alcohol cross-feeding between *Rahnella* and *Pseudomonas* in the co-culture (Figure 4). Proteins identified as differentially abundant in co-culture versus glucose monoculture are listed in Table S8; the complete results for all proteins identified in proteome measurements for this study are in Table S10.

## 4. Discussion

In this study, we have demonstrated that two *Populus* associates, *P. fluorescens* GM16 and *R. aquatilis OV744*, interact via cross-feeding of salicyl alcohol. In the larger context, *Rahnella* appears to act as a mediator of interaction between *Pseudomonas* and *Populus*. The association between these microbes and plants is mutually beneficial, as both *Rahnella* and *Pseudomonas* [6] display plant growth-promoting properties, and bacteria receive several benefits including availability of carbon sources. These nutrients are not always constant, with shifts between favorable and unfavorable sources [58,59,60,61,62,63].

Root exudation of phenolic glycosides by plants varies depending on a variety of biotic and abiotic factors, including developmental stage, season, pathogen challenge, and edaphic conditions [64,65]. Therefore, plant- and soil-associated bacteria that have evolved systems that can readily degrade phenolic glycosides have a fitness advantage in plant-related niches. For instance, bacteria-producing polyphenol associated enzymes can utilize polyphenols to improve persistence in gut regions [66]. *P. fluorescens* GM16 cannot utilize salicin, but its association with salicin-utilizing bacteria such as *Rahnella* could possibly mediate the *Populus* colonization process. *R. aquatilis OV744* might have been able to successfully colonize the *Populus* rhizosphere, because either (1) it could readily degrade salicin, or (2) it might have acquired this system over time, because of its association with *Populus* [67,68]. In *E. coli*, it has been proposed that mutations in the promotor region of silent *bgl* operon or in the genes encoding other glucosidases are required for the activation of salicin-degradation ability [23,69]. *Rahnella* possesses a *bgl*-like operon and most likely uses a similar mechanism of salicin degradation as was corroborated by this study. The intermediate of the proposed mechanism, salicin 6-phosphate, was not detected in the metabolomic analysis of supernatant of salicin cultures. This metabolite was also not detected in the metabolomic study involving intracellular metabolites, because salicin 6-phosphate is likely immediately consumed within the cell.

Salicin metabolism by *Rahnella* provides the cross-feeding metabolite, salicyl alcohol (Figure 1B), that can be utilized by *P. fluorescens* GM16 for growth. Salicyl alcohol is not generally considered a growth substrate. In fact, salicyl alcohol has been demonstrated to inhibit microbial growth [70]. Furthermore, the intermediates of the proposed salicyl alcohol pathway are also toxic to microbes including *Pseudomonas* species [71,72,73,74,75,76,77,78]. On the other hand, studies have demonstrated that a few strains of *Pseudomonas* are able to utilize not only benzyl alcohol and similar compounds, but also intermediates of the proposed pathway using distinct branches of naphthalene degradation channel [17,18,19,20,79]. *P. fluorescens* GM16 possesses a salicyl alcohol utilization pathway which requires several enzymes of the naphthalene degradation pathway. The salicyl alcohol degradation capability likely provides *P. fluorescens* GM16 a distinct advantage in colonizing *Populus* roots. Another possibility is that this pathway might be a result of evolutionary specialization, because of *Pseudomonas*’s association with salicin-degrading organisms in the root microbiome.

In this study, genomic data were validated by proteomics data that confirm the activity of the genes responsible for the transport and degradation of salicin. For instance, one of the probable proteins involved in salicin transport was EX29DRAFT_03584 based on genomic data (see Figure S3). The proteome comparison between glucose and salicin monocultures of *Rahnella* suggested that the protein could be a potential candidate based on differential abundance (Table S4), even though the absolute abundance in salicin monoculture was not very strong (Table S10). When the *Rahnella* proteome was analyzed in the co-culture dataset, however, the protein was not detected, lending less support to the involvement of EX29DRAFT_03584 in salicin transport (see Tables S8 and S10). However, the protein signal for EX29DRAFT_03584 being outcompeted, because of the presence of twice the number of proteins in co-culture cannot be ruled out. Somewhat more successfully, proteome data could be utilized to identify the likely proteins involved in each of the multiple reactions of the pathway. For instance, enzyme 3-oxoadipyl-CoA thiolase (EC: 2.3.1.174/2.3.1.174) responsible for the conversion of oxoadipyl-CoA to succinyl-CoA and acetyl-CoA is predicted to be carried out by multiple enzymes, but the proteome data suggests PMI19_04401 as a likely candidate.

The exact mechanism of export of salicyl alcohol, however, could not be characterized, because no prior knowledge of the exporters is available. Protein sequences of known exporters of related compounds, such as toluene [80,81,82,83], were queried against the *R. aquatilis OV744* genome, and these gene hits were matched against the proteome datasets. No conclusive result could be found from this analysis (Table S9), nor could the importer of salicyl alcohol in *P. fluorescens* GM16 be determined conclusively using the proteomics data. Therefore, at this point, we cannot predict the mechanism of salicyl alcohol uptake in *Pseudomonas*. It could very likely be a diffusion process, which is the case for a related compound 4-hydroxybenzoic acid [84]. It is also possible that the proteomics method was not able to detect the transporters with sufficient signal, because of its lower sensitivity to membrane proteins in general.

In co-culture, the proteomics and metabolite data provide direct evidence that this two-member pathway is utilized by both strains to grow cooperatively on salicin by cross-feeding salicyl alcohol. Since both strains are morphologically similar and because of the quick dilution of fluorescence, microscopic techniques were not feasible (data not shown) making qPCR the technique of choice to elucidate the growth of both strains in co-culture. Surprisingly, the data also showed that *Pseudomonas* was able to take over the growth in the co-culture as seen in Figure 3C. This is an interesting phenomenon because *Rahnella* is the provider of carbon source in the proposed unidirectional cross-feeding interaction. A similar growth dynamic has been previously reported [85]. The lower final copy count of *Rahnella* in co-culture could be attributed to two factors: (1) starting copy count numbers [86] and (2) competition between co-culture members [87]. First, we discovered that starting copy count of *Pseudomonas* in salicin co-culture was higher than that of *Rahnella* in co-culture which might provide growth advantage to *Pseudomonas*. Likewise, the starting copy count of *Rahnella* in salicin monoculture was higher than that in co-culture. Therefore, comparing copy count numbers between salicin monocultures and co-cultures for *Rahnella* was not informative. Second, the presence of *Pseudomonas* made the comparison more challenging. The soluble factors released by *Pseudomonas* did not appear to inhibit the growth of *Rahnella* (see Figure S7), but nothing conclusive can be assumed yet. It is reasonable to suggest that competition for resources between *Rahnella* and *Pseudomonas* causes a decrease in growth of *Rahnella* in co-culture [86]. More assays and analyses are required to determine the nature of interaction between *Rahnella* and *Pseudomonas* in salicin co-culture.

Laboratory and natural conditions might not be same. Moreover, these two strains were isolated from different niches in *Populus* (*Rahnella*-rhizosphere and *Pseudomonas*-endosphere) but they do co-exist with similar organisms in nature [37]. With the results from *in vitro* assays, we hypothesize that *Pseudomonas* is a selfish cheater, such that this interaction neither harms nor helps *Rahnella* in the natural system. Such “cheating” interactions have been widely reported in the literature [29,33,88]. Furthermore, another possible reason these two strains can co-exist in nature could be that both organisms benefit from being in contact with *Populus*.

**Figure 5:**
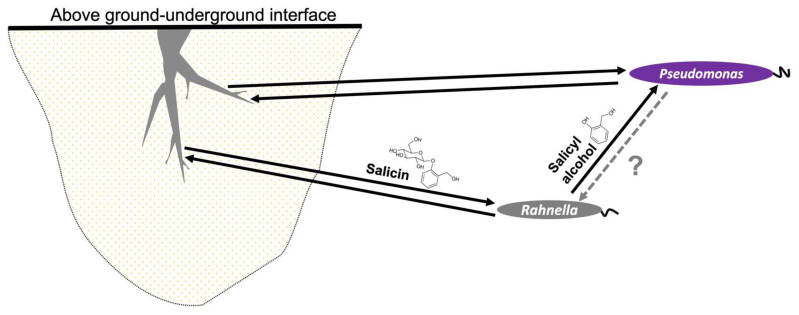
Proposed model of mechanism of microbial recruitment of *P. fluorescens* GM16 on *Populus* by the facilitator *R. aquatilis OV744* through PGs such as salicin. In the suggested model, plants provide PG carbon substrates to recruit bacteria. In the *Populus*–*Rahnella*–*Pseudomonas* model, *Rahnella* can utilize salicin produced by *Populus* and releases salicyl alcohol as a by-product. Salicyl alcohol is then utilized by *Pseudomonas*. In return, neither beneficial nor harmful activity has yet been observed. Both *Pseudomonas* and *Rahnella* have demonstrated plant-growth promoting properties suggesting mutually beneficial relationship between these microbes and *Populus*. In the figure, dark arrows suggest beneficial interaction and the grey, dotted arrow represents yet unknown interaction.

Our study has demonstrated a mechanism by which a PG such as salicin could be involved in microbial recruitment in the *Populus* microbiota. These compounds are used by salicin-degrading organisms, such as *Rahnella,* which utilizes a *bgl* system to consume salicin with subsequent release of salicyl alcohol as a by-product. The advantage of such a system is poorly understood, but one study has demonstrated that this aglycone molecule can act in bacterial defense by killing bacterivores [89]. We propose another possible advantage of this system, which is to attract other microorganisms toward the *Populus* microbiome, as suggested by chemotaxis assays (Figure S8). Therefore, we offer a model of a tripartite interkingdom relationship in which *Rahnella* acts as mediator between *Populus* and *Pseudomonas*, but only benefits from its association with *Populus*, not with *Pseudomonas* (Figure 5).

## 5. Conclusions

In summary, we utilized multiple approaches to demonstrate that salicin-degrading strains (such as *R. aquatilis OV744*) facilitate *Populus* colonization by organisms, such as *P. fluorescens* GM16, through cross-feeding of salicyl alcohol. Therefore, PGs could be used for attracting at least two different types of organisms into the *Populus* microbiome. The co-culture model used in this project could be further studied to understand the nature of interaction between the two microbial members. This study could be crucial in elucidating plant colonization by multiple bacteria and shedding light on the evolution of the relationship between *Populus* and its microbiome.

## Figures and Tables

**Table 1:** Proteins in a probable pathway of salicyl alcohol degradation were significantly upregulated in the proteome of *P. fluorescens* GM16 when grown on salicyl alcohol versus growth on glucose.

Locus Tag	Annotation	pfp ≤ 0.05 ^a^	log_2_[SalOH:Glu] ^b^
PMI19_01133	acyl-CoA reductase	Yes	6.2
PMI19_01134	aryl-alcohol dehydrogenase	Yes	6.1
PMI19_01136	salicylate hydroxylase	Yes	(up)
PMI19_01135	catechol 1,2-dioxygenase	Yes	(up)
PMI19_01184	catechol 1,2-dioxygenase	Yes	6.8
PMI19_03945	catechol 1,2-dioxygenase	No	(up)
PMI19_01186	muconate cycloisomerase	Yes	(up)
PMI19_03943	muconate cycloisomerase	-	ND
PMI19_01185	muconolactone delta-isomerase	Yes	(up)
PMI19_03944	muconolactone delta-isomerase	-	ND
PMI19_04396	3-oxoadipate enol-lactonase	Yes	(up)
PMI19_04846	3-oxoadipate enol-lactonase	-	ND
PMI19_01175	3-oxoadipate CoA-transferase, alpha subunit	Yes	(up)
PMI19_01176	3-oxoadipate CoA-transferase, beta subunit	No	(up)
PMI19_00175	acetyl-CoA acyltransferase	-	ND
PMI19_01217	3-ketoacyl-CoA thiolase	No	0.25
PMI19_03256	3-ketoacyl-CoA thiolase	No	0.73
PMI19_04401	3-oxoadipyl-CoA thiolase	Yes	4.6

Purple/blue/orange/yellow/light red/dark red font: proteins predicted to have similar function shown with same color font. ^a^ pfp values for Rank Product comparison of the two cultures values to evaluate significance of difference in NSAF values in salicyl alcohol culture versus glucose culture. ^b^ log_2_ of the ratio of NSAF values for salicyl alcohol (SalOH) versus glucose (Glu) cultures. For proteins not detected in glucose culture, the corresponding denominator in the NSAF ratio was only estimated. Therefore, those are assigned either “up” or “down”, shown in parentheses. ND: not detected in either treatment (in grey rows).

**Table 2:** Probable proteins involved in salicin degradation for *R. aquatilis OV744* were detected in proteome of salicin cultures, but not in glucose cultures.

Locus Tag	Annotation	pfp ≤ 0.05 ^a^	log_2_[Sal:Glu] ^b^
EX29DRAFT_02289	PTS system beta-glucoside-specific IIA component, Glc family (TC 4.A.1.2.2)/PTS system beta-glucoside-specific IIB component, Glc family (TC 4.A.1.2.2)/PTS system beta-glucoside-specific IIC component, Glc family (TC 4.A.1.2.2)	Yes	(up)
EX29DRAFT_02290	6-phospho-beta-glucosidase	Yes	(up)
EX29DRAFT_02291	transcriptional antiterminator, BglG family	-	ND

^a^ Percentage of false prediction (pfp) value for Rank Product comparison of the two cultures values to evaluate significance of difference in NSAF values in salicin culture versus glucose culture. ^b^ log_2_ of the ratio of NSAF values for salicin (Sal) cultures versus glucose (Glu) cultures. For proteins not detected in glucose culture, the corresponding denominator in the NSAF ratio was only estimated. Therefore, those are assigned either “up” or “down”, shown in parentheses. ND: not detected in either treatment (in grey row).

## Data Availability

The proteomics mass spectrometric output files in the original instrument vendor file format, Myrimatch search results, IDPicker analysis files, and the protein sequence file used for searches have been deposited to ProteomeXchange (http://proteomecentral.proteomexchange.org/cgi/GetDataset?ID=PXD015876, (accessed on 30 December 2022) dataset identifier PXD015876) via MassIVE (http://massive.ucsd.edu/ProteoSAFe/QueryPXD?id=PXD015876, (accessed on 30 December 2022) dataset identifier MSV000084467).

## Supplementary Materials

The following supporting information can be downloaded at: https://www.mdpi.com/article/10.3390/metabo13020140/s1, Figure S1: Growth delay of *P. fluorescens* GM16 in salicyl alcohol is not due to emergence of mutants; Figure S2: Growth curve analysis of *P. fluorescens* GM16 on key intermediates of proposed pathway; Figure S3: Possible GH1 enzyme encoding genes and their clusters in *R. aquatilis OV744*; Figure S4: Stable state of the co-culture demonstrates that *P. fluorescens* GM16 and *R. aquatilis OV744* grow in the ratio of ~2:1; Figure S5: Growth of *R. aquatilis OV744* in monoculture and co-culture in salicin minimal media; Figure S6: Salicyl alcohol does not significantly inhibit the growth of *R. aquatilis OV744*; Figure S7: *P. fluorescens* GM16 secretion does not affect the growth of *R. aquatilis OV744*; Figure S8: Chemotaxis assays demonstrate *P. fluorescens* GM16 is attracted towards salicyl alcohol; Table S1: Proposed genes in *P. fluorescens* GM16; Table S2: Proteomic comparison between glucose and salicyl alcohol cultures of *P. fluorescens* GM16; Table S3: Proposed genes in *R. aquatilis OV744*; Table S4: Proteomic comparison between glucose and salicin cultures of *R. aquatilis OV744*; Table S5: qPCR data to determine abundances *P. fluorescens* GM16 and *R. aquatilis OV744* in co-culture over time; Table S6: Stable state determination of the co-culture of P. *fluorescens* GM16 and *R. aquatilis OV744* over time; Table S7: Comparison of abundances of *R. aquatilis OV744* in glucose and salicin cultures over time; Table S8: Proteomic comparison between glucose monocultures and salicin co-culture of *R. aquatilis OV744* and *P. fluorescens* GM16; Table S9: Probable salicyl alcohol exporters in *R. aquatilis OV744*; Table S10: Complete list of proteins detected in proteome measurements with quantitative results.
