# Supplementary figures and images for: Mechanism for Utilization of the Populus-Derived Metabolite Salicin by a Pseudomonas—Rahnella Co-Culture
Source: Metabolites. 2023 Jan 17;13(2):140. doi: 10.3390/metabo13020140 (PMC9959693; doi:10.3390/metabo13020140)

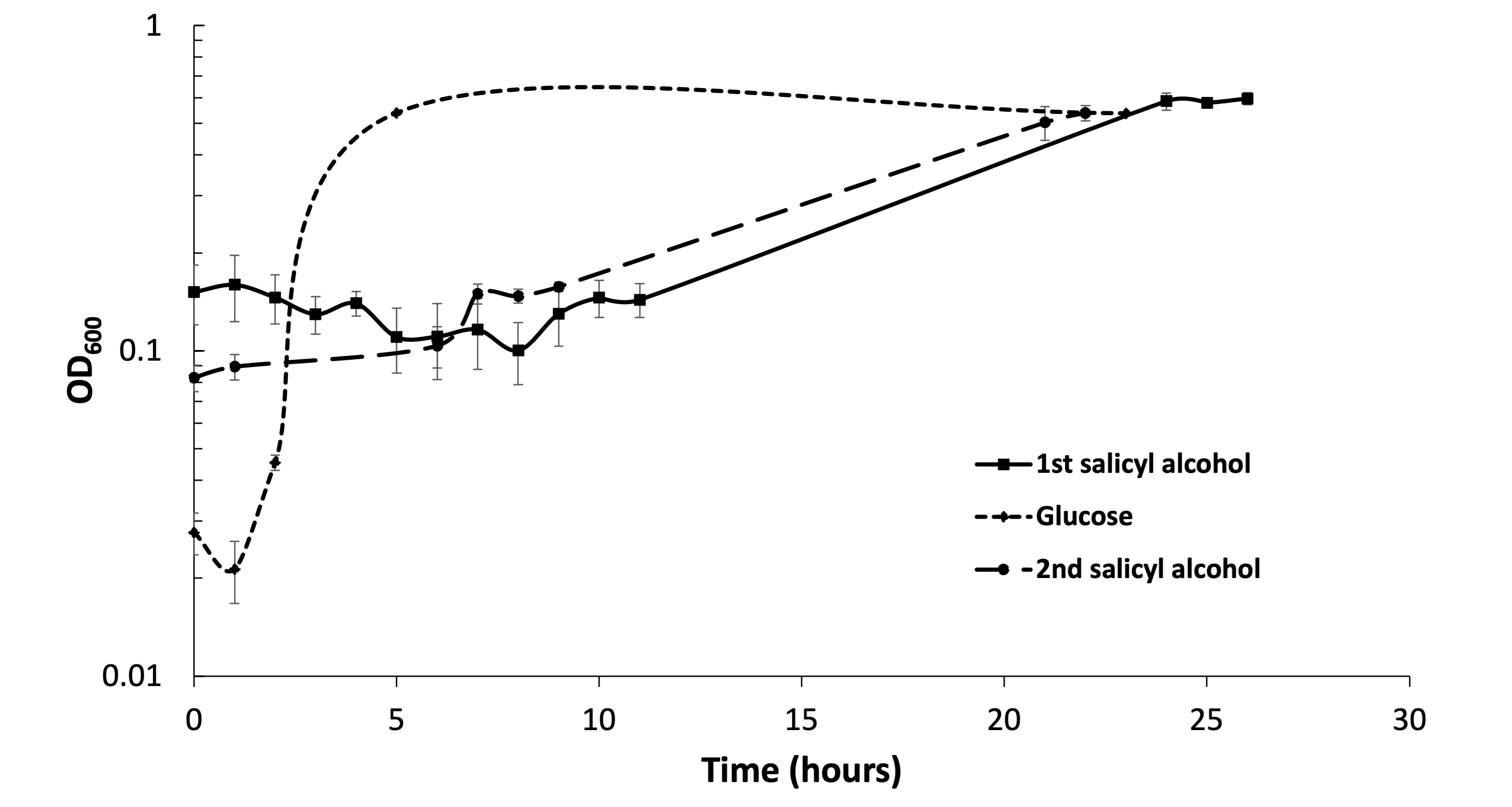

Supplement: Supplementary file 1 [file metabolites-13-00140-s001.zip › FigureS1.jpg]

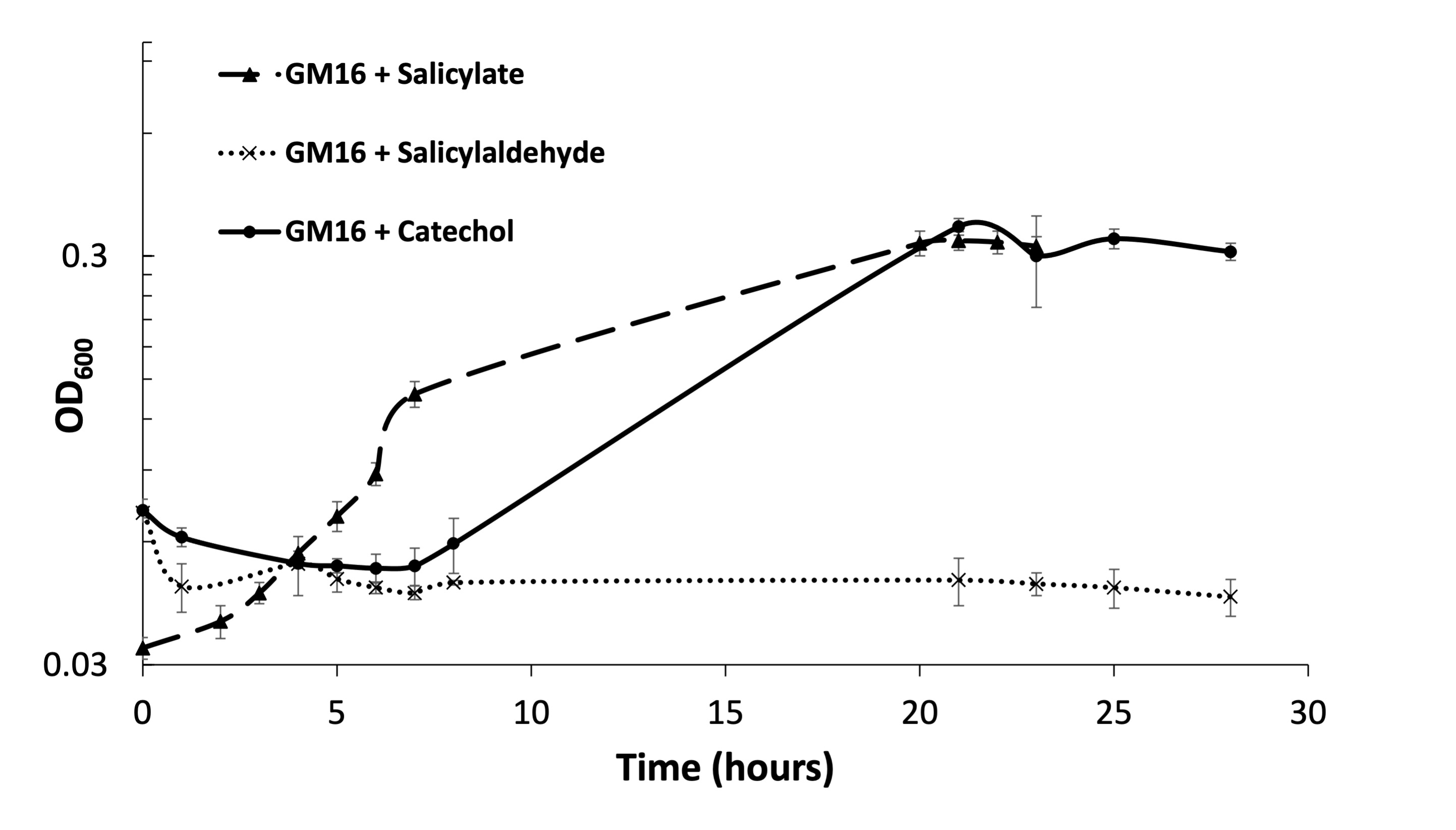

Supplement: Supplementary file 1 [file metabolites-13-00140-s001.zip › FigureS2.jpg]

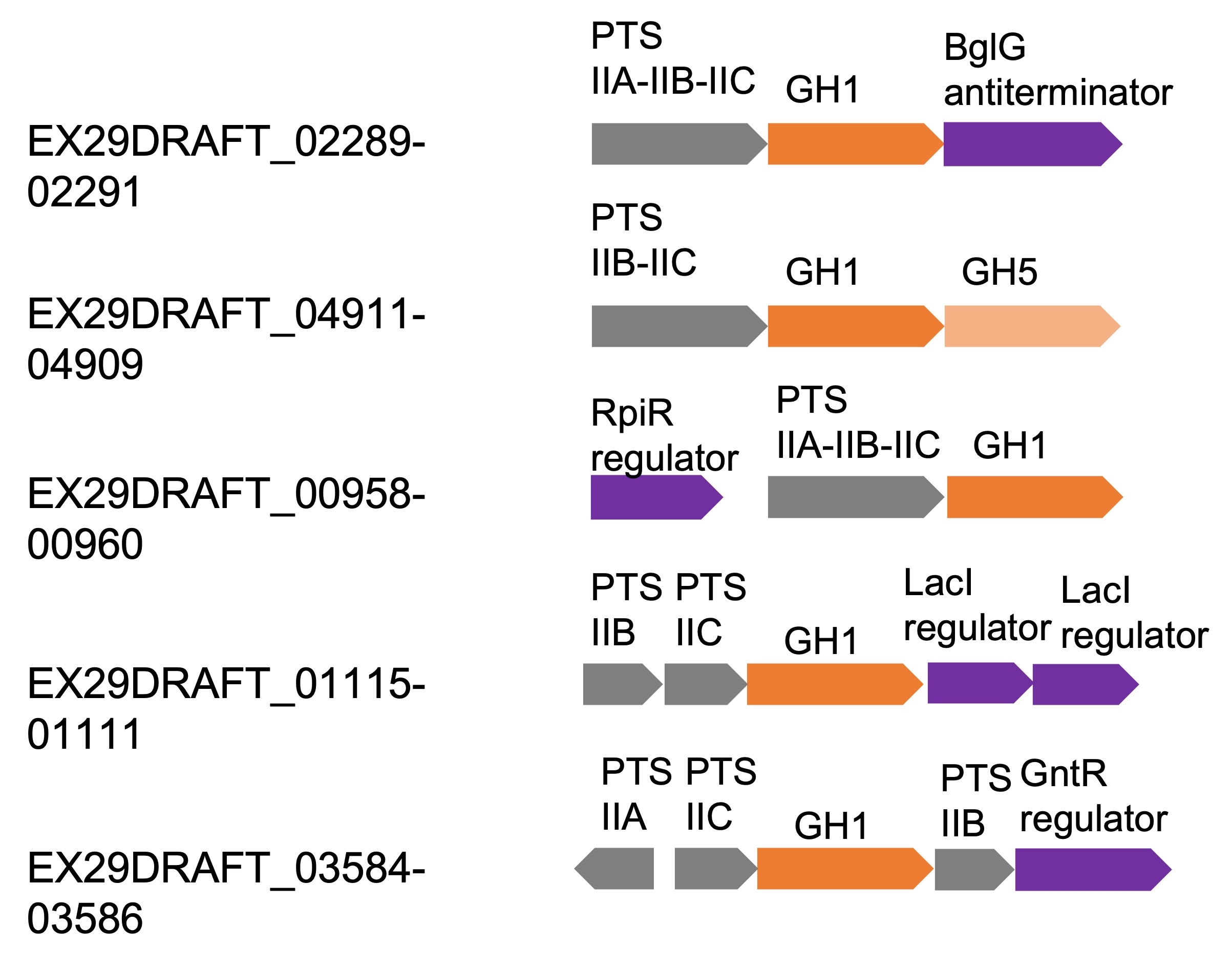

Supplement: Supplementary file 1 [file metabolites-13-00140-s001.zip › FigureS3.jpg]

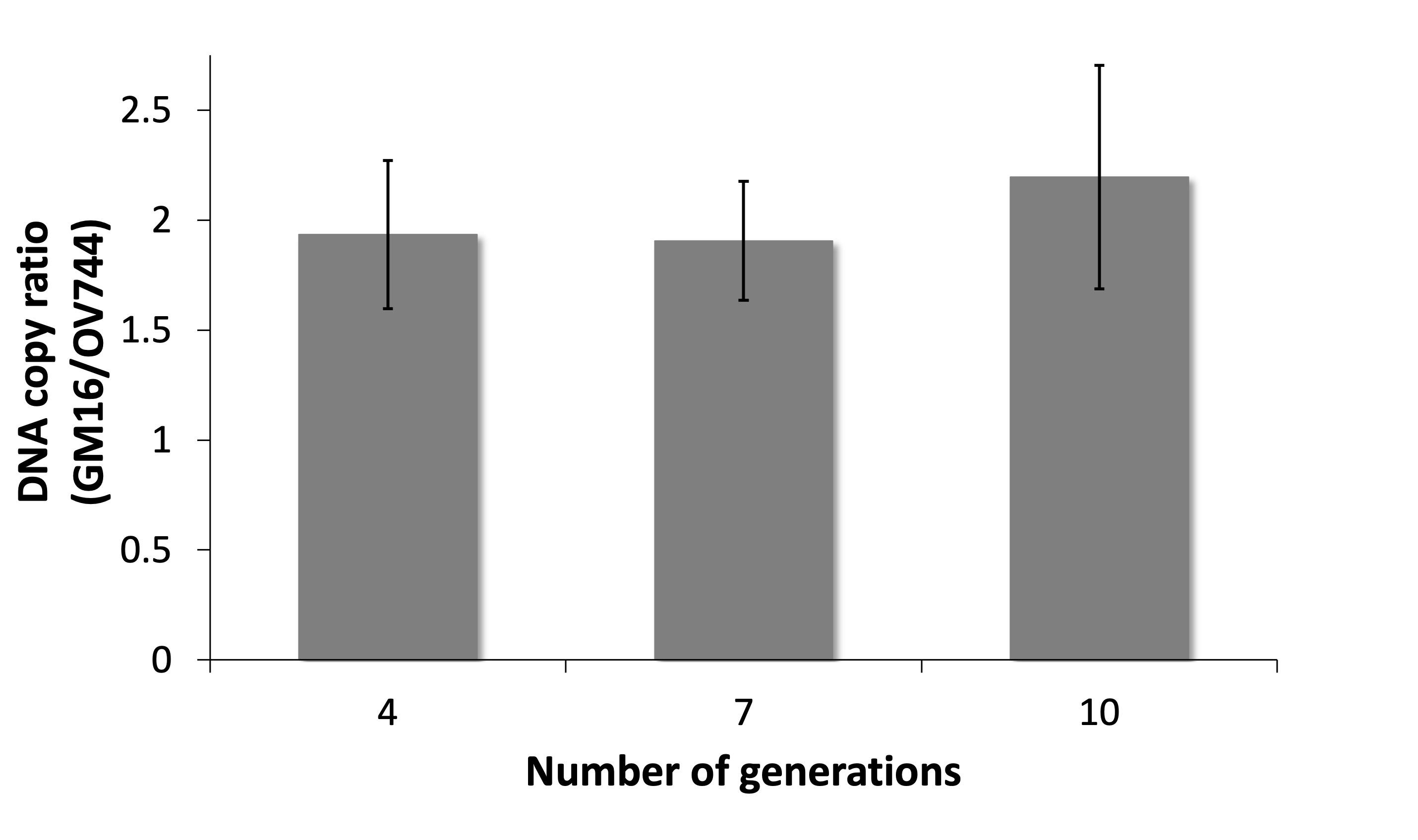

Supplement: Supplementary file 1 [file metabolites-13-00140-s001.zip › FigureS4.jpg]

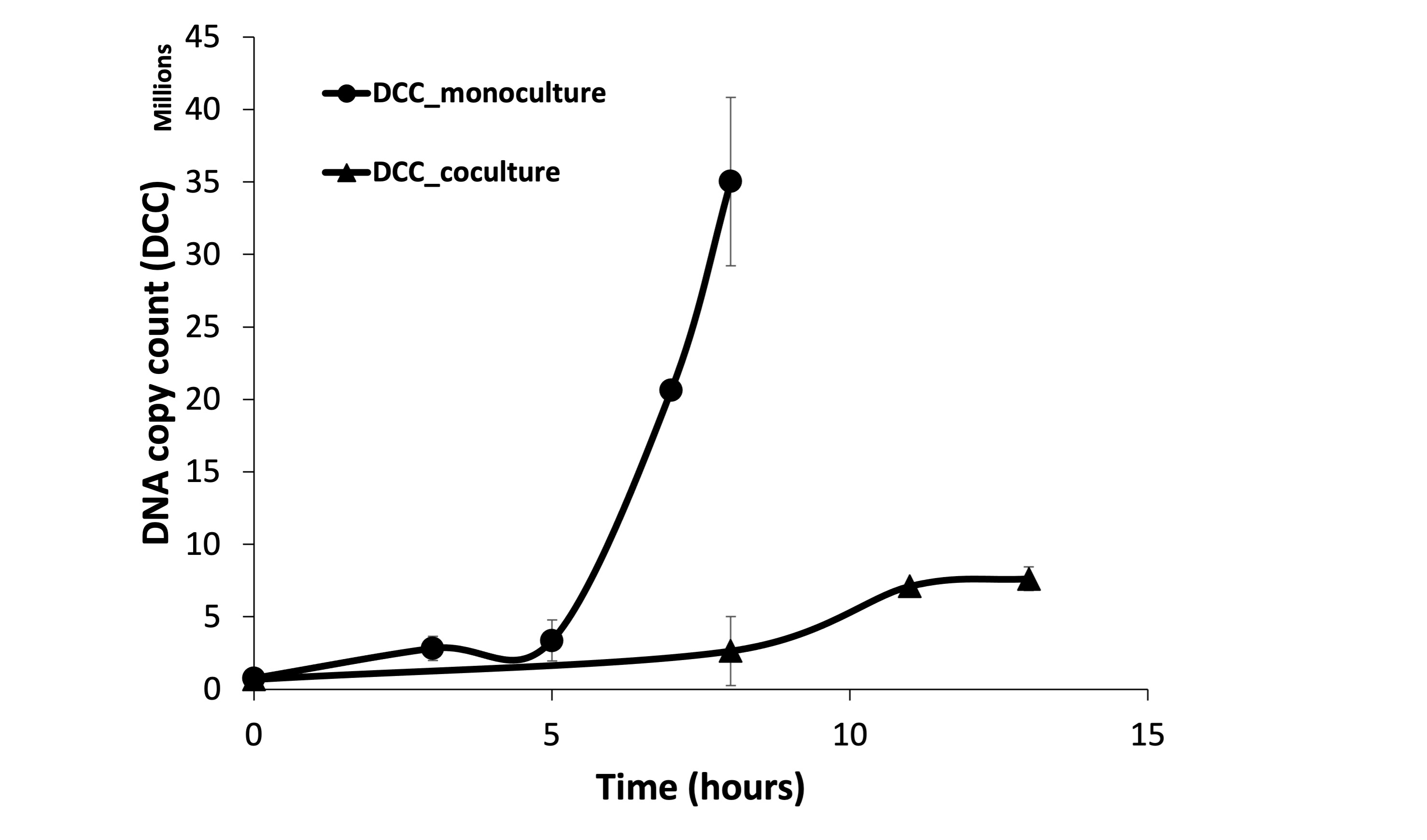

Supplement: Supplementary file 1 [file metabolites-13-00140-s001.zip › FigureS5.jpg]

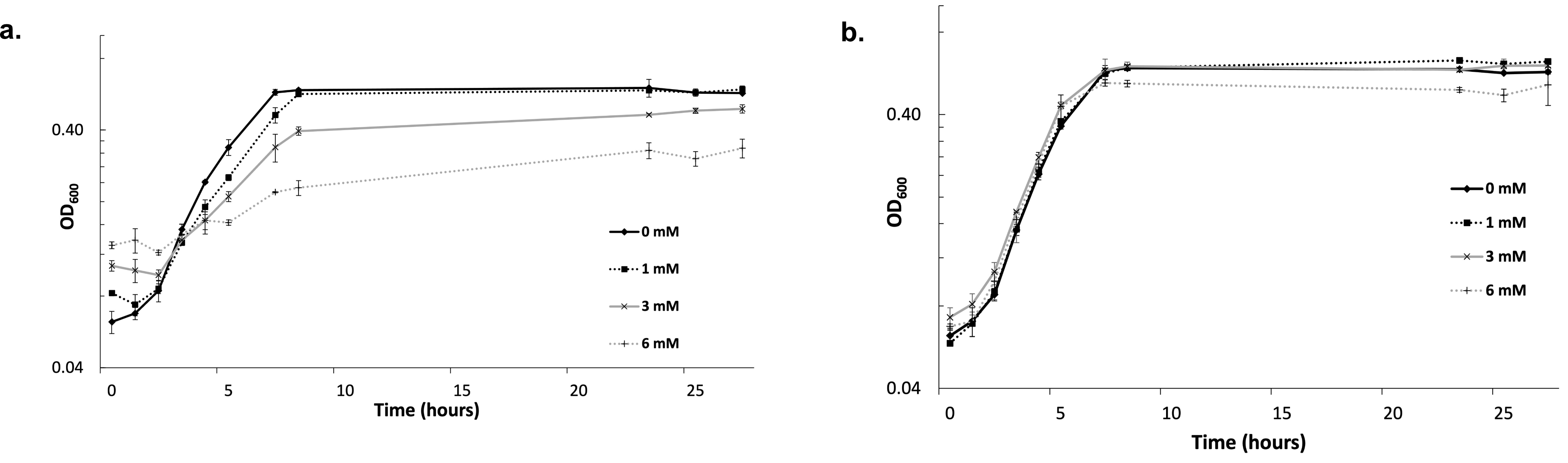

Supplement: Supplementary file 1 [file metabolites-13-00140-s001.zip › FigureS6.jpg]

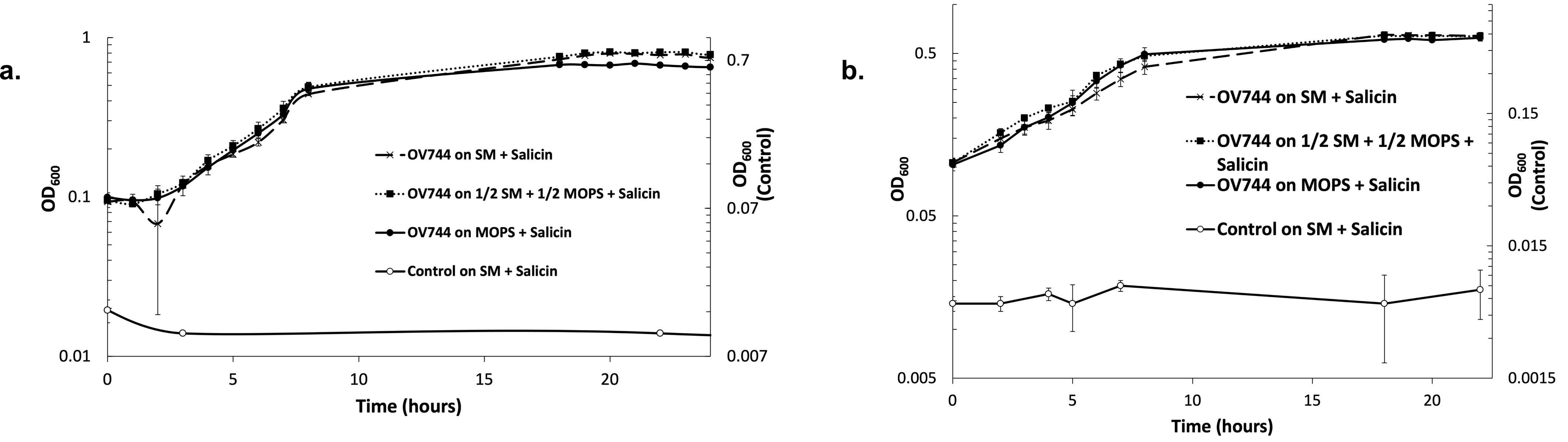

Supplement: Supplementary file 1 [file metabolites-13-00140-s001.zip › FigureS7.jpg]

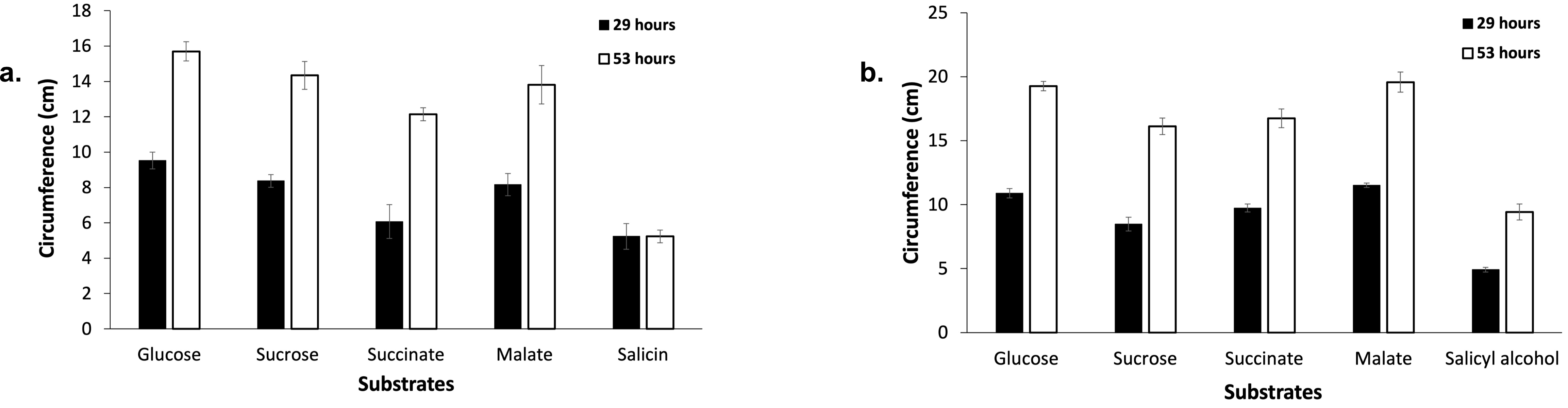

Supplement: Supplementary file 1 [file metabolites-13-00140-s001.zip › FigureS8.jpg]
